# Surveillance of the equine infectious anemia virus in Eastern and Central Saudi Arabia during 2014-2016

**DOI:** 10.14202/vetworld.2019.719-723

**Published:** 2019-05-29

**Authors:** Abdulmohsen Abdullah Alnaeem, Maged Gomaa Hemida

**Affiliations:** 1 Department of Clinical Studies, College of Veterinary Medicine King Faisal University, Saudi Arabia; 2 Department of Microbiology and Parasitology, College of Veterinary Medicine, King Faisal University, Saudi Arabia; 3 Department of Virology, Faculty of Veterinary Medicine, Kafrelsheikh University, Egypt

**Keywords:** enzyme-linked immunosorbent assay, equine infectious anemia virus, real-time polymerase chain reaction, Saudi Arabia

## Abstract

**Background:**

Equine infectious anemia virus (EIAV) is one of the most important threats to the equine industry globally. This is due to the poor performance of the affected horses, which requires euthanization of the infected animals upon the infection confirmation. Infected animals remain carriers throughout their life. EIAV infection has been reported in many parts of the world, including North America, Europe, Asia, and Africa. However, the EIAV status is never assessed in horses in the Gulf area, especially in the Kingdom of Saudi Arabia (KSA).

**Aim:**

This study aimed to perform molecular and serological surveillance among some horse populations in Eastern and Central Saudi Arabia.

**Materials and Methods:**

Sera and whole blood were collected from 361 horses and 19 donkeys from the eastern and central regions of Saudi Arabia during January 2014-December 2016. Sera were tested by the commercial EIAV enzyme-linked immunosorbent assay kits. Moreover, the collected blood samples were tested by the commercial real-time polymerase chain reaction kits.

**Results:**

Our serological surveillance revealed the absence of any antibodies against EIAV in the tested animals. Similar results were reported for the tested horses’ plasma. This study confirms the absence of EIAV in horses and donkeys from Eastern and Central Saudi Arabia during the tenure of the current study. However, careful monitoring of the EIAV is highly recommended to avoid the emergence of such a virus in the horse population in Saudi Arabia.

**Conclusion:**

To the best of our knowledge, this is the first EIAV surveillance conducted not only in Saudi Arabia but also in the Gulf area. This study confirms the absence of EIAV in the tested equine population in the eastern and central regions of Saudi Arabia during 2014-2016.

## Introduction

Equine infectious anemia virus (EIAV) is one of the most devastating and notifiable viral diseases of the family Equidae [1]. EIAV infection ranges from acute, subacute, and chronic infection. Acute infection in horses usually resulted in high morbidity and mortality rates up to 80% of the affected animals [2]. However, some chronically infected animals remain carriers throughout their life. These animals act as an amplifier and source of shedding the virus to other animals [3]. This may contribute to the sustainability of the EIAV infection in certain population or group of animals in certain geographical areas [4]. EIAV belongs to the family *Retroviridae* and genus *Lentivirus*. EIAV was reported in many parts of the world, including North America [5], South America [6], Europe [1], and Asia [7-11]. Unfortunately, no medication that treats EIAV-infected horses is available. However, some vaccination trials were tried to control the EIAV. In the 1970s, attenuated EIAV vaccines, including the donkey leukocyte-adapted strain (DLV121) and dermal cell-adapted strain (FDDV13), were produced by sequential serial passage of a virulent field isolate of EIAV (LN40) *in vivo* in donkeys (DV117), in primary donkey monocyte-derived macrophages, and in fetal donkey dermal cells.

The prevalence of EIAV in China was controlled by nationwide utilization of EIAV DLV121 [12,13]. The only control measures are to conduct survey-based monitoring for the EIAV among horse population. This is usually done through regular serological surveillances by the agar gel immunodiffusion (AGID) test and the enzyme-linked immunosorbent assay (ELISA) [14-17]. According to the OIE recommendations, screening of sera from animals by ELISA should be done first, then confirmation of the EIAV-positive animals by the AGID [18]. AGID considered the serological test of choice for testing the exposure history of horse population to the EIAV, Recombinant ELISA based on the P26 gene acts as a screening test for the EIAV. The current situation of the EIAV in the Gulf area is not clear yet. Serological surveillance was conducted in Sultanate of Oman in 2011 confirmed the absence of EIAV in some horse populations across the country using both ELISA and AGID test [19]. However, in the Kingdom of Saudi Arabia (KSA), nothing is known about the status of EIAV in horses and donkeys across the country.

The current study was designed to evaluate the status of some horse and donkey population in two major regions in the KSA. Meanwhile, some serum and whole blood from horses and donkeys were collected from the eastern and central regions of KSA, which have the most intensive horse population across the country. The collected sera were tested by the commercial ELISA kits. The whole blood was tested by the commercial quantitative polymerase chain reaction (q-PCR). To our knowledge, this is the first study investigating the molecular and serological prevalence of the EIAV among horses and donkeys in the eastern and central regions of Saudi Arabia.

## Materials and Methods

### Ethical approval

All animal experiments and sample collection were conducted as per the King Abdulaziz City for Science and Technology guidelines. This animal utilization protocol was amended by the King Faisal’s University Animal Ethics and the National Committee of Bioethics.

### Study area

**Figure-1:**
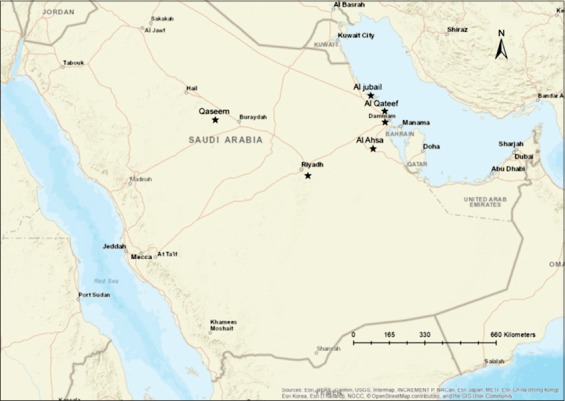
Geographical map of Saudi Arabia showing the distribution of the analyzed horses and donkeys. Map of the Kingdom of Saudi Arabia showing the location of the target horse population under study (2014-2016). Black asterisks mark the target horse’s population.

The current study focused on two major regions from Saudi Arabia with dense equine populations, the eastern and the central regions. These two regions are homes of the majority of equine population in the KSA. Several horse stables were selected on Al-Hasa, Dammam, Al-Qatif, and Al-Jubail in the eastern region. Furthermore, other horse populations from Riyadh and Al-Qassim regions representing the central region were sampled (Figure-1 and Table-1). These samples were collected during the time from January 2014 until November 2016. These samples were collected throughout the year representing the four seasons. Different categories of horse’s management were included in the sampling. Some animals used to take part in local horse races and shows, and others used to help farmers as heavy-duty animals. Some animals were in close contact with other animals such as camels, cattle, sheep, goat, and chickens. Samples were collected from at least 30% of each target population per each locality. The surveillance was conducted as a part of large study evaluating the status of some equine population in Eastern and Central Saudi Arabia to the most common viral diseases.

**Table-1:** Summary of the collected samples, their geographical locations, and results.

N	City	No. of tested animals	Horses				No. of tested animals	Donkeys			
	
			ELISA		q-PCR			ELISA		q-PCR	
			
			+Ve	−Ve	+Ve	−Ve		+Ve	−Ve	+Ve	−Ve
1	H	60	0	60	0	60	9	0	9	0	9
2	Qt	130	0	130	0	130	7	0	7	0	7
3	D	52	0	52	0	52	3	0	3	0	3
4	J	53	0	53	0	53	0	0	0	0	0
5	R	47	0	47	0	47	0	0	0	0	0
6	Qs	19	0	19	0	19	0	0	0	0	0
Total		361	0	361	0	361	19	0	19	0	19

D=Dammam, H=Al-Hasa, J=Al-Jubail, Qs=Al-Qassim, Qt=Al-Qatif, ELISA=Enzyme-linked immunosorbent assay, q-PCR=Quantitative polymerase chain reaction

### Sample collection and processing

#### Serum samples

Three hundred and eighty serum samples were collected from horses and donkeys in various regions in Eastern and Central Saudi Arabia (Table-1 and Figure-1). All animals used in this study were in good health, showing no obvious clinical signs of any disease. The selected animals included both sexes and various categories of ages. Moreover, the animals used in these surveillances represented different management systems such as racing, farming, and sentinel herds. None of these animals were vaccinated against EIAV. Blood samples were collected by venepuncture from the jugular vein and kept overnight at 4°C. The collected blood samples were centrifuged at 5000 RPM for 5 min, and the serum was transferred to new tubes. Serum samples were heat-inactivated to remove the non-specific inhibitors at 56°C for 30 min. Sera were allowed to cool down to the room temperature. Then, the sera were stored at –20°C for further testing.

#### Whole blood

The 380 whole blood samples out of the 361 from horses and 19 from donkeys sampled were collected via venipuncture. The blood samples were collected in the tubes containing ethylenediaminetetraacetic acid (EDTA), and gentle shaking was applied to mix the whole blood with the EDTA. The samples were transferred on ice to the authors’ laboratory. The whole blood was processed as previously described [1]. Specifically, the whole blood was centrifuged at 5000 RPM for 5 min at 4°C. Next, the buffy coat was carefully collected by pipetting. Finally, these buffy coats were kept at –80°C for downstream testing using molecular techniques.

#### ELISA

The collected serum samples were tested in duplicate using the commercially available ELISA kits (ID Screen^®^ ID Screen^®^ Equine Infectious Anemia Double Antigen ELISA kit, Cat No: EIAED-4P, Innovative Diagnostics, Montpelier, France) according to the manufacturer’s instructions and as previously described elsewhere [19]. The ELISA plate’s optical density (OD) was measured at 405 nm by the Synergy TM Mx, BioTek microplate ELISA reader (Winooski, USA). Interpretation of the tested samples was performed through the following formula: (Blocking percentage (BP%) = (Negative OD−Sample OD)/(Negative OD−Positive OD)). Any sample was considered positive when BP% percentage was ≥50%.

#### Ribonucleic acid (RNA) extraction

The total viral nucleic acids (NAs) were extracted from the collected whole blood by the QIA-amp Viral Mini Kit (Qiagen, Hilden, Germany) as per the manufacturer’s instructions. The viral NA concentration was assessed by the Nanodrop machine (Thermo Scientific NanoDrop 2000, Applied Biosystems), and the NA samples were stored at −80^○^C until testing.

#### Equine infectious anemia virus real-time reverse transcriptase-q-PCR

The extracted viral NAs from the collected whole blood were evaluated for the EIAV proviral DNA with the commercial real-time PCR kits (EIAV-genesig^®^ Easy Kit, Path-EIAV-Standard, PrimerDesign Ltd., Chandler’s Ford, United Kingdom). The real-time PCR technique was conducted according to the manufacturer’s instructions. Initially, the DNAs were denatured as previously described [20]. Briefly, 15-µl reaction was prepared including 10 µl of Oasig^™^ qPCR master mix, 1 µl of EIAV probe, and 4 µl of DNAase/RNase-free water. 15 µL of master mix was transferred to each well of the real-time PCR plates. To the master mix, 5 µl of each NA sample was added to the assigned well. The PCR reaction conditions were reverse transcription at 55^○^C for 10 min, activation of the enzymes at 95^○^C for 2 min, denaturation at 95^○^C for 10 s, and then data collection at 60^○^C for 60 s. These parameters were repeated for 50 cycles. The test was valid when the positive control generated a cycle threshold (Ct) between cycles 16 and 23. The tested sample was considered positive if the Ct <35. This test was carried out in the ABI 7500 thermal cycler (Applied Biosystems Inc., California, USA).

## Results

### Evaluation of the antibody response of horses to EIAV in Eastern and Central Saudi Arabia

A total of 380 serum samples were tested from horses and donkeys in Eastern and Central Saudi Arabia for the presence of EIAV antibodies by the commercial ELISA kits as described above. All the tested serum samples were negative for the EIAV antibodies (Table-1).

### Molecular surveillance of EIAV in Eastern and Central Saudi Arabia

A total of 380 whole blood samples collected from horses and donkeys in Eastern and Central KSA were tested by the commercial real-time PCR kits. Our data showing all the tested blood samples were negative for the NA of the EIAV (Table-1).

## Discussion

EIAV remains one of the top-listed OIE notifiable viral diseases of the family Equidae [18]. One of the most important problems in the identification of the EIAV-infected animals is the lack of obvious clinical signs in most of the cases [1]. The infected horse may act as a source of infection to other animals in the same premises through flies biting [1]. One serological surveillance was conducted on some horses in Sultanate Oman in early 2011 [19], this study reported the absence of the EIAV in the tested horse premises at that time. However, there were no other EIAV reports from other countries in the Gulf area, especially the KSA. There are almost 28546 horses as per the last records from the King Abdulaziz Arabian Horses Center (DIRAB). Both the central and eastern regions of the kingdom include at least one one-sixth of these horse population. The main goal of the current study was to assess the status of the EIAV among several horse and donkey populations in both Eastern and Central KSA. Our serosurveillance data confirmed the absence of any detectable antibodies for the EIAV in the sera of the tested horses and donkeys during the study (Table-1). Based on the given information by the commercial ELISA company, it had sensitivity and specificity of 100% [21]. That is why our serum samples were not tested further by the AGID test as another confirmatory test. A similar approach was applied for the seroprevalence in many other studies in India, Turkey, and Spain [21-23]. Similarly, all the tested blood samples were negative for the EIAV by the q-PCR (Table-1). In conclusion, we acknowledge the absence of horses’ population from Eastern and Central KSA during 2014-2016. However, regular monitoring of horse population across the country is recommended to ensure the continuous freedom of the country from the EIAV.

## Conclusion and Recommendation

It is concluded that horses and donkeys tested from Eastern and Central Saudi Arabia did not show any detectable antibodies for the EIAV during tenure of the current study (2014-2016). Continuous surveillance of the equine population for the EIV is highly recommended not only in the Gulf area but also worldwide.

## Authors’ Contributions

MGH and AAA collected specimens, performed the laboratory techniques, data analysis, and drafted and revised the manuscript. Both authors read and approved the final manuscript.

## References

[1] Gaudaire D, Lecouturier F, Ponçon N, Morilland E, Laugier C, Zientara S, Hans A (2018). Molecular characterization of equine infectious anaemia virus from a major outbreak in southeastern France. Transbound. Emerg. Dis.

[2] Motie A (1986). An outbreak of suspected equine infectious anemia in Guyana. Br. Vet. J.

[3] Craigo J.K, Sturgeon T.J, Cook S.J, Issel C.J, Leroux C, Montelaro R.C (2006). Apparent elimination of EIAV ancestral species in a long-term inapparent carrier. Virology.

[4] Knowles N.J, Shirazi M.H.N, Wadsworth J, Swabey K.G, Stirling J.M, Statham R.J, Li Y, Hutchings G.H, Ferris N.P, Parlak U, Ozyörük F, Sumption K.J, King D.P, Paton D.J (2009). Recent spread of a new strain (A-Iran-05) of foot-and-mouth disease virus type A in the Middle East. Transbound. Emerg. Dis.

[5] Nagarajan M.M, Simard C (2007). Gag genetic heterogeneity of equine infectious anemia virus (EIAV) in naturally infected horses in Canada. Virus Res.

[6] Oliveira F.G, Cook R.F, Naves J.H.F, Oliveira C.H.S, Diniz R.S, Freitas F.J.C, Lima J.M, Sakamoto S.M, Leite R.C, Issel C.J, Reis J.K.P (2017). Equine infectious anemia prevalence in feral donkeys from Northeast Brazil. Prev. Vet. Med.

[7] Bolfa P, Jeon I, Loftis A, Leslie T, Marchi S, Sithole F, Beck C, Lecollinet S, Zientara S, Hans A, Issel C.J (2017). Detection of West Nile virus and other common equine viruses in three locations from the Leeward Islands, West Indies. Acta Trop.

[8] Sharav T, Konnai S, Ochirkhuu N, Ts E.O, Mekata H, Sakoda Y, Umemura T, Murata S, Chultemdorj T, Ohashi K (2017). Detection and molecular characterization of equine infectious anemia virus in Mongolian horses. J. Vet. Med. Sci.

[9] Uppal P.K, Yadav M.P (1989). Occurrence of equine infectious anemia in India. Vet. Rec.

[10] Wang H.N, Rao D, Fu X.Q, Hu M.M, Dong J.G (2018). Equine infectious anemia virus in China. Oncotarget.

[11] Yapklc O, Yavru S, Kale M, Bulut O, Simşek A, Sahna K.C (2007). An investigation of equine infectious anemia infection in the central Anatolia region of Turkey. J. S. Afr. Vet. Assoc.

[12] Wang X.F, Lin Y.Z, Li Q, Liu Q, Zhao W.W, Du C, Chen J, Wang X, Zhou J.H (2016). Genetic evolution during the development of an attenuated EIAV vaccine. Retrovirology.

[13] Wang X.F, Liu Q, Wang Y.H, Wang S, Chen J, Lin Y.Z, Ma J, Zhou J.H, Wang X (2018). Characterization of equine infectious anemia virus long terminal repeat quasispecies *in vitro* and *in vivo*. J. Virol.

[14] Issel C.J, Cook R.F (1993). A review of techniques for the serologic diagnosis of equine infectious anemia. J. Vet. Diagn. Invest.

[15] Pare J, Simard C (2004). Comparison of commercial enzyme-linked immunosorbent assays and agar gel immunodiffusion tests for the serodiagnosis of equine infectious anemia. Can. J. Vet. Res.

[16] Piza A.S, Pereira A.R, Terreran M.T, Mozzer O, Tanuri A, Brandão P.E, Richtzenhain L.J (2007). Serodiagnosis of equine infectious anemia by agar gel immunodiffusion and ELISA using a recombinant p26 viral protein expressed in *Escherichia coli* as antigen. Prev. Vet. Med.

[17] Ricotti S, Garcia M.I, Veaute C, Bailat A, Lucca E, Cook R.F, Cook S.J, Soutullo A (2016). Serologically silent, occult equine infectious anemia virus (EIAV) infections in horses. Vet. Microbiol.

[18] OIE (2012). Manual od Diagnostic Tests and Vaccines for Terrestrial Animals (Mammals, Birds, and Bees).

[19] Body N, Al-Rawahi A, Hussain M, AL-Lamki K, Al-Habsy S, Almaawali M, Alrawahi Q (2011). Sero-survey of equine infectious anemia in the Sultanate of Oman during 2007-2009. Pak. Vet. J.

[20] Dong J.B, Zhu W, Cook F.R, Goto Y, Horii Y, Haga T (2012). Development of a nested PCR assay to detect equine infectious anemia proviral DNA from peripheral blood of naturally infected horses. Arch. Virol.

[21] Cruz F, Fores P, Ireland J, Moreno M.A, Newton R (2015). Freedom from equine infectious anemia virus infection in Spanish purebred horses. Vet. Rec. Open.

[22] Albayrak H, Ozan E (2010). Serosurveillance for equine infectious anemia in the Ardahan province of Turkey. Trop. Anim. Health Prod.

[23] Malik P, Singha H, Goyal S.K, Khurana S.K, Kumar R, Virmani N, Shanmugasundaram K, Pandey S.B, Kant R, Singh B.K, Singh R.K (2013). Sero-surveillance of equine infectious anemia virus in equines in India during more than a decade (1999-2012). Indian J. Virol.

